# Causal relationships between hippocampal volumetric traits and the risk of Alzheimer’s disease: a Mendelian randomization study

**DOI:** 10.1093/braincomms/fcaf030

**Published:** 2025-01-23

**Authors:** Lining Guo, Yayuan Chen, Zuhao Sun, Jiaxuan Zhao, Jia Yao, Zhihui Zhang, Minghuan Lei, Ying Zhai, Jinglei Xu, Yurong Jiang, Ying Wang, Hui Xue, Mengge Liu, Feng Liu

**Affiliations:** Department of Radiology and Tianjin Key Laboratory of Functional Imaging and Tianjin Institute of Radiology, Tianjin Medical University General Hospital, 30052 Tianjin, China; Department of Radiology and Tianjin Key Laboratory of Functional Imaging and Tianjin Institute of Radiology, Tianjin Medical University General Hospital, 30052 Tianjin, China; Department of Radiology and Tianjin Key Laboratory of Functional Imaging and Tianjin Institute of Radiology, Tianjin Medical University General Hospital, 30052 Tianjin, China; Department of Radiology and Tianjin Key Laboratory of Functional Imaging and Tianjin Institute of Radiology, Tianjin Medical University General Hospital, 30052 Tianjin, China; Department of Radiology and Tianjin Key Laboratory of Functional Imaging and Tianjin Institute of Radiology, Tianjin Medical University General Hospital, 30052 Tianjin, China; Department of Radiology and Tianjin Key Laboratory of Functional Imaging and Tianjin Institute of Radiology, Tianjin Medical University General Hospital, 30052 Tianjin, China; Department of Radiology and Tianjin Key Laboratory of Functional Imaging and Tianjin Institute of Radiology, Tianjin Medical University General Hospital, 30052 Tianjin, China; Department of Radiology and Tianjin Key Laboratory of Functional Imaging and Tianjin Institute of Radiology, Tianjin Medical University General Hospital, 30052 Tianjin, China; Department of Radiology and Tianjin Key Laboratory of Functional Imaging and Tianjin Institute of Radiology, Tianjin Medical University General Hospital, 30052 Tianjin, China; Department of Radiology and Tianjin Key Laboratory of Functional Imaging and Tianjin Institute of Radiology, Tianjin Medical University General Hospital, 30052 Tianjin, China; Department of Radiology and Tianjin Key Laboratory of Functional Imaging and Tianjin Institute of Radiology, Tianjin Medical University General Hospital, 30052 Tianjin, China; Department of Radiology and Tianjin Key Laboratory of Functional Imaging and Tianjin Institute of Radiology, Tianjin Medical University General Hospital, 30052 Tianjin, China; Department of Radiology and Tianjin Key Laboratory of Functional Imaging and Tianjin Institute of Radiology, Tianjin Medical University General Hospital, 30052 Tianjin, China; Department of Radiology and Tianjin Key Laboratory of Functional Imaging and Tianjin Institute of Radiology, Tianjin Medical University General Hospital, 30052 Tianjin, China

**Keywords:** Alzheimer’s disease, hippocampal volumetric traits, Mendelian randomization, causal relationships, neuroimaging

## Abstract

Alzheimer’s disease, a common and progressive neurodegenerative disorder, is associated with alterations in hippocampal volume, as revealed by neuroimaging research. However, the causal links between the volumes of the hippocampus and its subfield structures with Alzheimer’s disease remain unknown. A genetic correlation analysis using linkage disequilibrium score regression was conducted to identify hippocampal volumetric traits linked to Alzheimer’s disease. Following this, to examine the causal links between Alzheimer’s disease and hippocampal volumetric traits, we applied a two-sample Mendelian randomization approach, utilizing a bidirectional framework. Seven hippocampal volumetric traits were found as genetically correlated with Alzheimer’s disease in the genetic correlation analysis and were then included in the Mendelian randomization analyses. Inverse variance weighted Mendelian randomization analyses revealed that increased volumes in the left whole hippocampus, left hippocampal body, right presubiculum head and right cornu ammonis 1 head were causally related to higher risks of Alzheimer’s disease. Conversely, a higher risk of Alzheimer’s disease was causally associated with decreased volumes of the left hippocampal body and left whole hippocampus. These results were validated through other Mendelian randomization approaches and sensitivity analysis. Our findings uncover bidirectional causal relationships between Alzheimer’s disease and hippocampal volumetric traits, suggesting not only the potential significance of these traits in predicting Alzheimer’s disease but also the reciprocal influence of Alzheimer’s disease on hippocampal volumes.

## Teaser

Guo *et al*. report bidirectional causal relationships between hippocampal volumetric traits and Alzheimer’s disease using Mendelian randomization. Their findings suggest increased hippocampal volume may raise Alzheimer’s disease risk, while higher disease risk is linked to reduced hippocampal volume, offering insights into the reciprocal effects of Alzheimer’s on hippocampal structures.

## Graphical Abstract

**graphical abstract:**
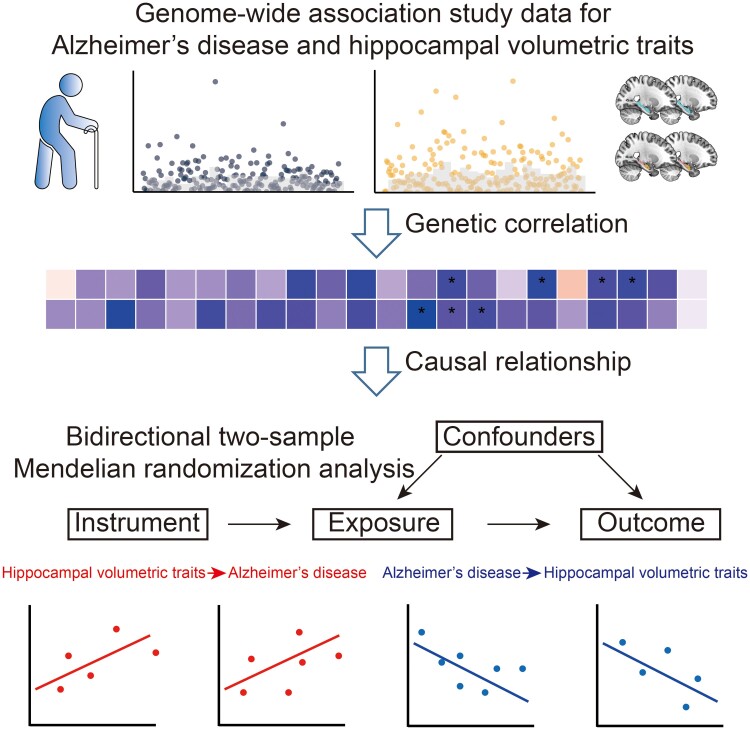

## Introduction

Alzheimer’s disease stands as a formidable public health concern, with its burden increasing as the global population ages.^1^ In 2022, around 55 million individuals globally were affected by dementia, with projections indicating this figure will rise to 132 million by 2050, with Alzheimer’s disease being the leading cause.^2^ Characterized by progressive cognitive decline,^3^ Alzheimer’s disease exacts a substantial burden on individuals, families and healthcare systems.^4^ The economic impact is equally staggering, with the global cost of dementia expected to surpass 2 trillion US dollars annually by 2030.^2^ As the search for effective prevention and intervention strategies intensifies, understanding the intricate relationships between neuroanatomical structures and Alzheimer’s disease risk becomes paramount.

The hippocampus, a vital structure nestled within the medial temporal lobe, has emerged as a focal point in the study of Alzheimer’s disease.^5,6^ Its pivotal role in memory consolidation and retrieval implicates the hippocampus in the pathophysiology of Alzheimer’s disease,^7^ where memory-related functions are particularly compromised.^8^ Neuroimaging studies consistently highlight alterations in hippocampal volume among individuals with Alzheimer’s disease, reflecting structural changes that may underpin cognitive decline.^9^ However, the causal link between hippocampal volumetric traits and the likelihood of developing Alzheimer’s disease continues to be an active area of research. On one hand, observational studies report inconsistent changes in hippocampal volume among individuals with Alzheimer’s disease with some indicating an increase^10-12^ and others a decrease,^13-15^ adding complexity to our understanding of this relationship. Due to the non-randomized nature of observational studies, challenges such as reverse causation, confounding and selection bias impede the establishment of causal relationships.^16^ On the other hand, it remains uncertain whether changes in hippocampal volume are a cause or a consequence of Alzheimer’s disease.^9,17^

Beyond its fundamental structure, the hippocampus reveals a diverse landscape, with distinct subregions characterized by unique cytoarchitectures.^18^ This diversity adds a layer of complexity to the exploration of hippocampal involvement in Alzheimer’s disease.^19^ Notably, the analysis of hippocampal subfields has emerged as a promising avenue in Alzheimer’s disease research. Studies emphasize localized atrophy, particularly in the CA1 subfield, during the early stages of Alzheimer’s disease occurring before the widespread atrophy observed in the dementia stage.^20,21^ Advances in neuroimaging techniques provide researchers with the opportunity to investigate these subregions,^22^ identifying subtle variations in structural integrity that may carry distinct implications for Alzheimer’s disease, further enhancing our understanding of the intricate relationship between hippocampal changes and Alzheimer’s disease.

In exploring the causal links between the hippocampus, its subfields and Alzheimer’s disease, our study employs the innovative framework of Mendelian randomization (MR). MR leverages naturally occurring genetic variants as instrumental variables (IVs), providing a quasi-experimental design to investigate causal relationships between an exposure and an outcome.^23,24^ The strength of this method stems from its capacity to effectively mitigate reverse causality and confounding factors, which are commonly encountered in observational studies, thus offering a more robust basis for causal inference.^25^ Moreover, recent years have witnessed an explosion of information on brain disorders and neuroimaging traits through genome-wide association studies (GWAS).^26-30^ This wealth of genetic data serves as a rich source for identifying relevant genetic variants pivotal in our MR analysis. In the context of our study, MR enables us to explore the causal pathways linking hippocampal volumes and Alzheimer’s disease risk, shedding light on whether variations in hippocampal structure contribute to the development of Alzheimer’s disease or if Alzheimer’s disease-related genetic factors influence hippocampal morphology.

## Materials and methods

### Data source

**Figure 1:**
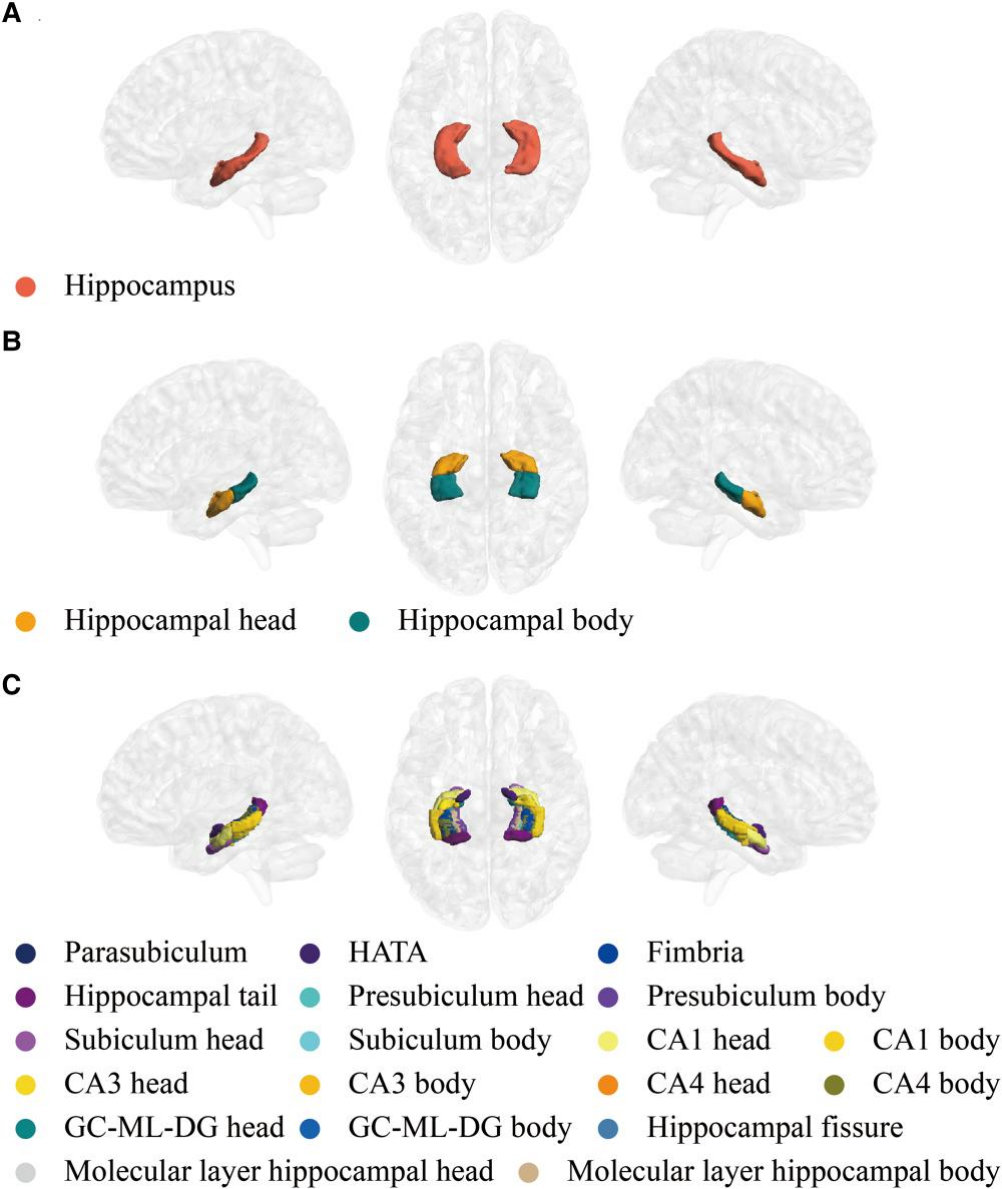
**The spatial distributions of the 44 hippocampal traits.** The hippocampus traits analysed in this study include the (**A**) whole hippocampus, (**B**) hippocampal head and body and (**C**) subfield structures. CA, cornu ammonis; GC-ML-DG, granule cell layer and molecular layer of dentate gyrus; HATA, hippocampus–amygdala transition area.

The GWAS summary statistics of hippocampal volumetric traits encompass a cohort of 33 224 individuals of European descent (17 411 females and 15 813) sourced from the UK Biobank (https://open.win.ox.ac.uk/ukbiobank/big40/).^31^ Hippocampal volumetric traits were segmented by FreeSurfer v7.0^32,33^ (https://surfer.nmr.mgh.harvard.edu/), covering 19 subfields on each side. These subfields encompass the hippocampus–amygdala transition area, parasubiculum, fimbria, tail, fissure and head and body of the subiculum, presubiculum, CA1, CA3, CA4, granule cell layer and molecular layer of the dentate gyrus, and molecular layer. Additionally, the bilateral hippocampal head, hippocampal body and whole hippocampus were included, resulting in a total of 44 traits for hippocampus and subfield volumes (Fig. 1). For all hippocampal volumetric traits, linear association tests were used in the GWAS, with careful control for covariates. In line with the approach described in the original study, this included regressing out head size, age, sex and other relevant factors.^31^ Specifically, covariates were removed using a comprehensive set of variables, including the 40 principal components of population genetics, along with additional covariates such as head motion during functional MRI, imaging centre, scanner table position and slow drifts related to scan dates. To ensure the clarity of the GWAS, all recommended covariates were adjusted for, as outlined in the study by Alfaro-Almagro *et al*.^34^ The same automatic selection thresholds were applied to higher-order (non-linear and interaction) covariates. Due to differences in the study samples, the original set of 602 confounds was reduced to 597 in this analysis to match the current data set.

The summary GWAS data for Alzheimer’s disease were drawn from a large-scale meta-analysis that included 90 338 Alzheimer’s disease cases and 1 036 225 controls of European ancestry (https://ctg.cncr.nl/software/summary_statistics), consolidated from 13 cohorts.^35^ The meta-analysis was conducted employing mv-GWAMA (https://github.com/Kyoko-wtnb/mvGWAMA), a method that weights by sample size, originally developed by Jansen *et al*.^36^ Specifically, the meta-analysis involved 39 918 cases and 358 140 controls after excluding data from the 23andMe and UK Biobank. The 23andMe data were excluded due to availability restrictions (3807 cases and 359 839 controls), while the UK Biobank data were excluded to prevent overlap with the hippocampal volume GWAS data set (46 613 cases and 318 246 controls), as overlapping samples between the Alzheimer’s disease and hippocampal volume data sets could introduce bias in MR analyses. Each of the included original studies reported receiving approval from local ethics committees, and each study ensured that informed consent was provided by all participants prior to their inclusion.

### Statistical analysis

#### Genetic correlation analysis

Genetic correlation refers to the extent to which genetic factors influence the covariance between two traits. In this study, the genetic correlation between hippocampal volumetric traits and Alzheimer’s disease was calculated using linkage disequilibrium score regression (https://github.com/bulik/ldsc/). This method estimates the shared genetic architecture by assessing how much the genetic variants associated with one trait (e.g. hippocampal volume) are also associated with another trait (e.g. Alzheimer’s disease). Precomputed linkage disequilibrium scores were obtained from the 1000 Genomes European reference panel, with the major histocompatibility complex region excluded due to its complex linkage disequilibrium structure (chr6: 28 477 797–33 448 354). Hippocampal volumetric traits showing a significance of *P* < 0.05 were selected for inclusion in the subsequent MR analysis.

#### Two-sample MR analysis

The MR analysis relies on three essential assumptions: (i) genetic variants must exhibit a strong association with the exposure; (ii) these IVs must not be linked to any biasing factors that could influence the association between the exposure and outcome; and (iii) the IVs should impact the outcome risk solely through their effect on the exposure. To investigate the causal link between hippocampal volumetric traits and Alzheimer’s disease, we performed bidirectional two-sample MR analyses, where ‘exposure’ refers to the genetic variants associated with either hippocampal volumetric traits or Alzheimer’s disease, depending on the direction of the analysis, while ‘outcome’ refers to the trait hypothesized to be influenced by the exposure. In one direction, hippocampal volumetric traits serve as the exposure, and Alzheimer’s disease risk is the outcome, allowing us to investigate whether variations in hippocampal volume affect the risk of developing Alzheimer’s disease. In the reverse analysis, Alzheimer’s disease risk is the exposure, and hippocampal volumetric traits are the outcome, helping us determine whether a genetic predisposition to Alzheimer’s disease leads to changes in hippocampal volume.

To conduct the MR analysis, we began by selecting IVs from the GWAS summary data for exposure, using a significance threshold of *P* < 5 × 10^−^⁸. Linkage disequilibrium clumping was then performed with an *r*^2^ cut-off value of 0.001 and a 10 000 kb sliding window. Where IVs for exposure were not available in the outcome data sets, they were substituted with proxy IVs (*r*² > 0.8), identified using the 1000 Genomes European reference data. Next, the effect alleles of these IVs were harmonized across both the exposure and outcome data sets to ensure consistent allele coding. Palindromic IVs with moderate allele frequencies were excluded, and IVs exhibiting pleiotropy identified through the heterogeneity test using RadialMR were subsequently discarded (*P* < 0.05).^37^ Following that, Steiger filtering was performed to remove IVs that explained more variance in the outcome than in the exposure.^38^ Finally, the *F*-statistic was computed to evaluate the potential presence of weak instrument bias among the retained IVs,^39^ and instruments with an *F*-statistic below 10 were excluded.

Given its high statistical power, the inverse variance weighted (IVW) method was used as the primary approach to evaluate the bidirectional relationship between hippocampal volumetric traits and Alzheimer’s disease.^40^ Since the IVW method can be influenced by pleiotropy and invalid IVs,^41^ we employed four additional MR techniques to evaluate the robustness of our results: MR-Egger, weighted mode, MR robust adjusted profile score and weighted median, to assess the robustness of our findings. In particular, MR robust adjusted profile score adjusts for both systematic and individual pleiotropy by incorporating overdispersion and applying robust loss functions, ensuring reliable results even when many weak instruments are present.^42^ The MR-Egger method permits pleiotropic effects on all variants and provides a consistent causal effect estimate, assuming weaker instrument strength and no direct effects (InSIDE assumption).^43^ The weighted-mode method groups IVs according to their causal impact values and calculates the causal effect using the largest group of IVs, yielding a reliable estimate when this largest group consists of valid IVs.^44^ Lastly, The weighted median approach provides a consistent causal effect estimate if at least half of the IVs used are valid.^41^ To correct for multiple comparisons, the results were adjusted using the Benjamini and Hochberg procedure for false discovery rate, with a significance threshold of *P* < 0.05.

To ensure the robustness of our MR results, the following analysis was carried out. First, the MR analysis was conducted again to mitigate the impact of potential confounders that might affect the findings. In this study, socioeconomic status was regarded as a confounder, as reported by previous studies to affect both hippocampal volumetric traits and Alzheimer’s disease.^45-47^ Accordingly, GWAS summary statistics on average total household income before tax, educational attainment and Townsend Deprivation Index at recruitment were obtained from previous literature.^48,49^ The IVs significantly associated with these confounders were then removed before conducting MR analysis, with the threshold set at Bonferroni-corrected *P* < 0.05. Subsequently, heterogeneity among the IVs was examined by applying Rücker’s *Q*-statistic for MR-Egger and Cochran’s *Q*-statistic for the IVW method. Third, to assess the presence of directional horizontal pleiotropy, MR-Egger regression was subsequently performed. A significant deviation from 0 in the intercept term would suggest the existence of directional pleiotropic bias. Fourth, to detect possible horizontal pleiotropy, we applied the Mendelian Randomization Pleiotropy Residual Sum and Outlier (MR-PRESSO) global test. If pleiotropy was found to be significant, the outlier detection step of MR-PRESSO was performed to identify any IVs that were outliers. Following the removal of identified outliers, MR estimates were recalculated to eliminate the detected pleiotropy. Finally, a leave-one-out analysis was conducted to determine if any individual IV had a disproportionate influence on the estimate or introduced bias. The significance level for the second, third and fourth analyses was defined as *P* < 0.05.

All of the previously mentioned MR analysis was performed utilizing the *R* packages *TwoSampleMR*^50^ (version 0.5.6), *RadialMR*^37^ (version 1.0), *mr.raps*^42^ (version 0.2) and *MRPRESSO*^51^ (version 1.0).

## Results

### Genetic correlation

Genetic correlations between 44 hippocampal volumetric traits and Alzheimer’s disease were investigated. Seven traits were identified as having nominal significance with Alzheimer’s disease, with correlation values ranging from −0.21 to −0.16 (Supplementary Fig. 1 and Supplementary Table 1).

### MR results

**Figure 2:**
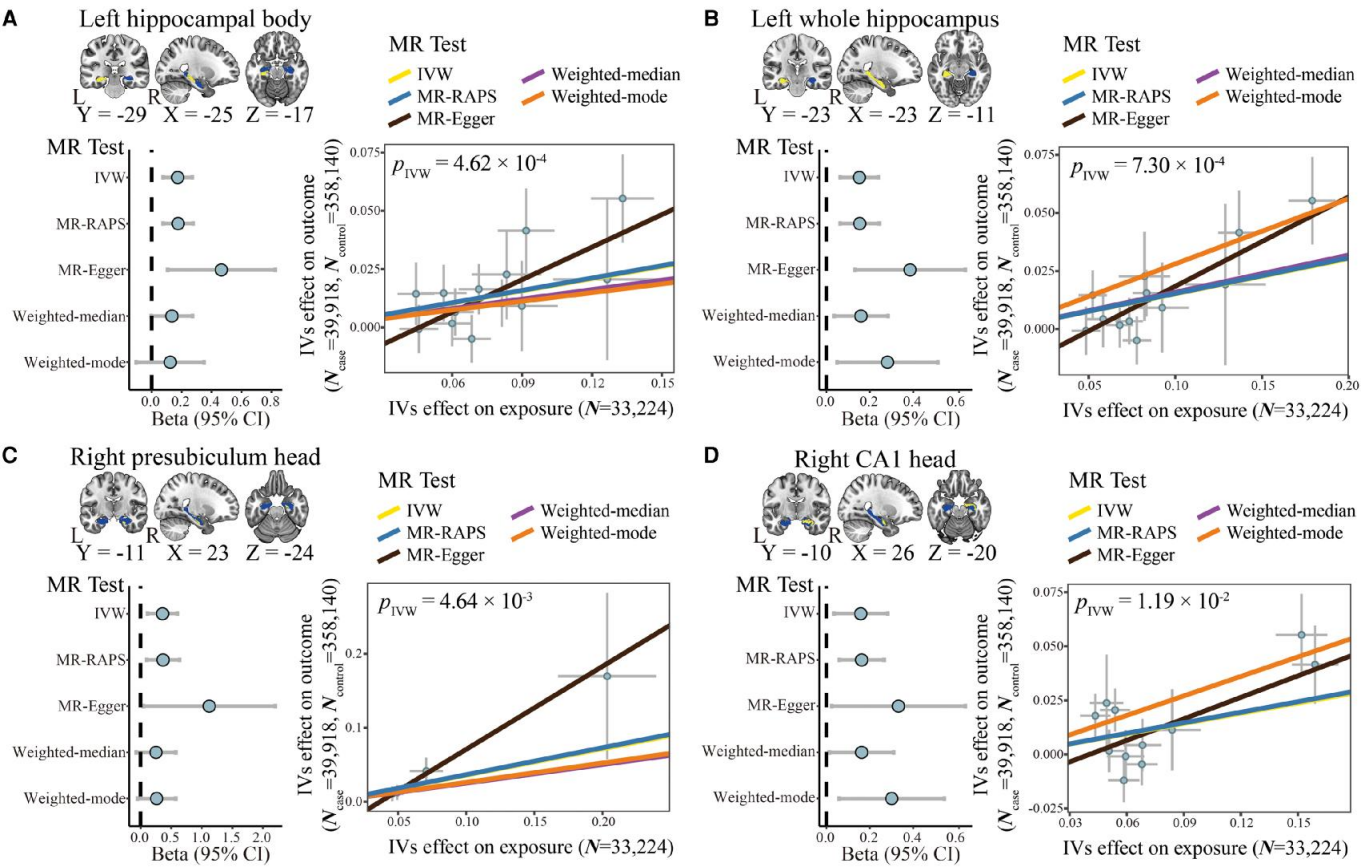
**Two-sample MR analysis of the causal effect of hippocampal volumetric traits on the risk of Alzheimer’s disease.** (**A–D**) The left panels show MR results for the causal effect of four hippocampal volumetric traits on Alzheimer’s disease. In the brain image (upper-left), the bilateral whole hippocampus is shown, with the regions representing the areas of interest in the hippocampal volumetric traits highlighted. The *x*-axis of the forest plot represents the causal effect size, and the *y*-axis indicated five different MR approaches (lower left). The right panels show scatter plots of the associations of four hippocampal volumetric traits with Alzheimer’s disease, with each dot represents an instrument, and the error bars at each dot represent the 95% CI. The *x*-axis represents the instrument effect on exposure (i.e. hippocampal volumetric traits), and the *y*-axis represents the instrument effect on outcome (i.e. Alzheimer’s disease). (**A**) Left hippocampal body, (**B**) left whole hippocampus, (**C**) right presubiculum head and (**D**) right CA1 head. CA, cornu ammonis; CI, confidence interval; IVs, instrumental variables; IVW, inverse variance weighted; L, left; MR, Mendelian randomization; MR-RAPS, MR robust adjusted profile score; *N*, number of participants; R, right.

Among the seven hippocampal volumetric traits genetically correlated with Alzheimer’s disease, four exhibit a causal impact on the development of Alzheimer’s disease (Fig. 2; Supplementary Table 2). These traits include the whole left hippocampus, the left hippocampal body, the right presubiculum head and the right CA1 head. Utilizing the IVW method, it was revealed that an increased volume of the left whole hippocampus causally influences the risk of Alzheimer’s disease, with a calculated odds ratio (*OR*) of 1.16 [95% confidence interval (CI) from 1.07 to 1.27, *P* = 7.30 × 10^−4^]. Similar results were obtained through alternative MR methods. Additionally, an augmented left hippocampal body volume demonstrated a significant causal effect on an increased risk of Alzheimer’s disease (*OR*_IVW_ = 1.19, 95% CI ranging from 1.08 to 1.31, *P* = 4.62 × 10^−4^), with comparable estimates from other MR methods. Likewise, an elevated volume of the right presubiculum head was causally linked to a higher risk of Alzheimer’s disease (*OR*_IVW_ = 1.43, 95% CI from 1.12 to 1.83, *P* = 4.64 × 10^−3^). Moreover, an increased volume of the right CA1 head was associated with an increased risk of Alzheimer’s disease (*OR*_IVW_ = 1.17, 95% CI from 1.04 to 1.33, *P* = 1.19 × 10^−2^).

**Figure 3:**
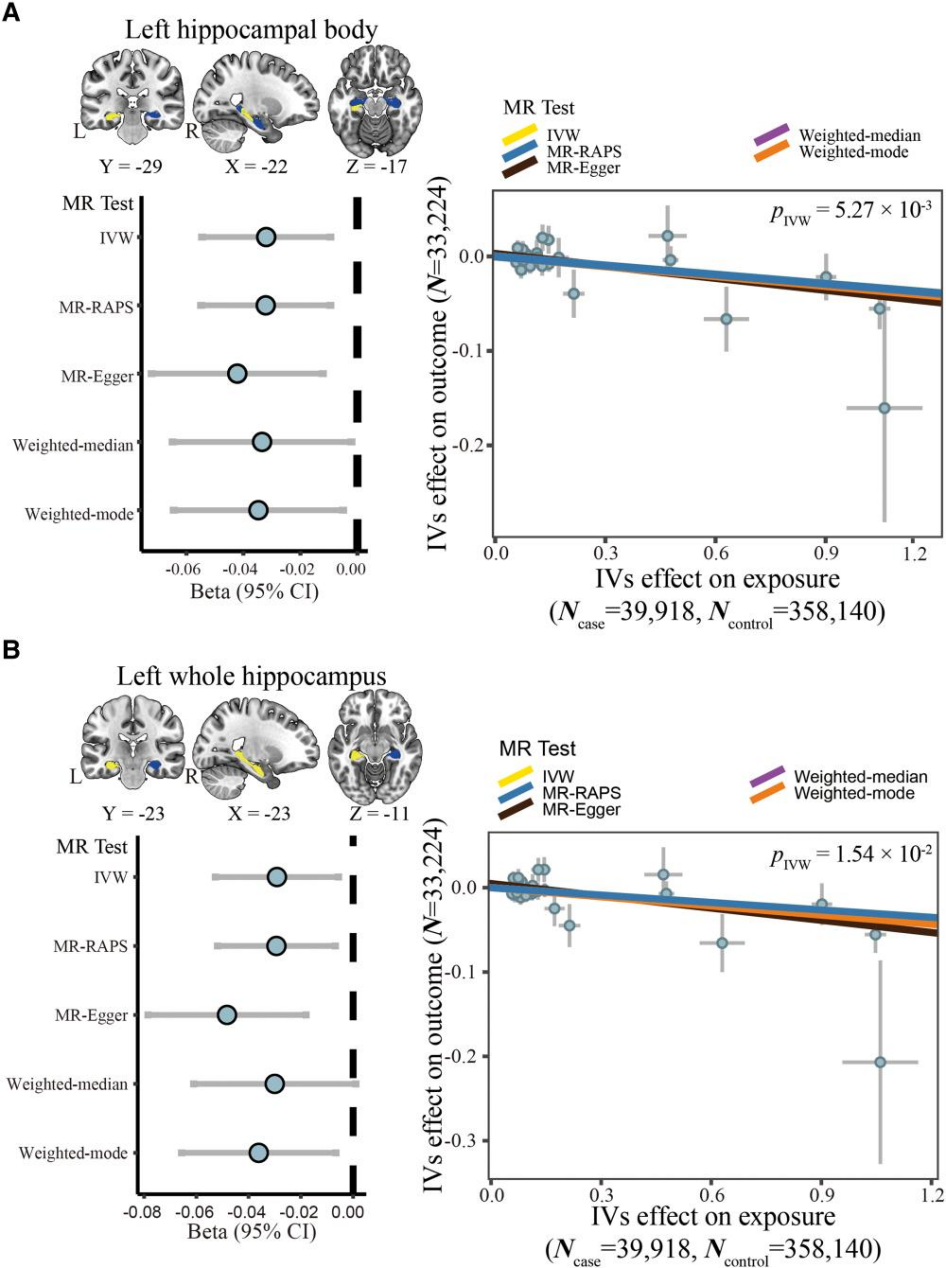
**Two-sample MR analysis of the causal effect of Alzheimer’s disease on hippocampal volumetric traits.** (**A**, **B**) The left panels show MR results for the causal effect of two hippocampal volumetric traits on Alzheimer’s disease. In the brain image (upper-left), the bilateral whole hippocampus is shown, with the regions representing the areas of interest in the hippocampal volumetric traits highlighted. The *x*-axis of the forest plot represents the causal effect size, and the *y*-axis indicated five different MR approaches (lower left). The right panels show scatter plots of the associations of two hippocampal volumetric traits with Alzheimer’s disease, with each dot represents an instrument, and the error bars at each dot represent the 95% CI. The *x*-axis represents the instrument effect on exposure (i.e. Alzheimer’s disease), and the *y*-axis represents the instrument effect on outcome (i.e. hippocampal volumetric traits). (**A**) Left hippocampal body and (**B**) left whole hippocampus. CI, confidence interval; IVs, instrumental variables; IVW, inverse variance weighted; L, left; MR, Mendelian randomization; MR-RAPS, MR robust adjusted profile score; *N*, number of participants; R, right.

Furthermore, it was observed that Alzheimer’s disease exerts a causal influence on two of the seven hippocampal volumetric traits (Fig. 3; Supplementary Table 3). Specifically, a higher risk of Alzheimer’s disease was causally related to a reduced volume of the left hippocampal body (*β*_IVW_ = −0.03, 95% CI −0.05 to −0.01, *P* = 5.27 × 10^−3^) and a decreased volume in the whole left hippocampus (*β*_IVW_ = −0.03, 95% CI −0.05 to −0.01, *P* = 1.54 × 10^−2^).

### Sensitivity analysis

The robustness of our findings was systematically examined through sensitivity analysis, reaffirming the consistency and reliability of our primary results. Controlling for potential confounding factors, including socioeconomic status, was executed in the MR analysis, ensuring the integrity of the results by removing IVs significantly associated with confounders (Supplementary Tables 4 and 5). Moreover, the absence of significant heterogeneity among IVs, as indicated by Rücker’s *Q* and Cochran’s *Q*-statistics, strengthened the validity of our results; both MR-PRESSO global test and MR-Egger regression showed no indication of directional horizontal pleiotropy (Supplementary Table 6). Furthermore, the leave-one-out analysis further validated the results, indicating that no single instrument had an undue influence on the findings (Supplementary Fig. 2).

## Discussion

This study is the first to undertake a comprehensive investigation of the causal relationship between hippocampal volumetric traits and Alzheimer’s disease. MR analysis reveals that four traits exhibit a significant causal impact on Alzheimer’s risk, encompassing the whole left hippocampus, left hippocampal body, right presubiculum head and right CA1 head. Additionally, our study unveils a reciprocal relationship, demonstrating that Alzheimer’s disease exerts a causal influence on the left hippocampal body and the whole left hippocampus. These findings, supported by alternative MR methods, advance our understanding of the complex relationships between hippocampal structure and Alzheimer’s disease, emphasizing the importance of specific subfield volumes in influencing disease risk.

The MR results present compelling evidence that four hippocampal traits exert a causal impact on the development of Alzheimer’s disease. Specifically, this study identified the significant causal effect of increased volumes of the whole left hippocampus, left hippocampal body, right presubiculum head and the right CA1 head on a higher risk of Alzheimer’s disease. Although the study does not explicitly outline the underlying mechanisms, it aligns with existing research indicating that structural changes, such as increased hippocampal volumes, may function as markers or outcomes of pathological processes linked to Alzheimer’s disease,^52^ which could encompass neuroinflammation, synaptic dysfunction or early neurodegenerative changes. However, it is important to note that previous literature has consistently reported lower hippocampal volumes in patients with mild cognitive impairment or subjective cognitive decline,^53,54^ both of which are often considered early precursors to Alzheimer’s disease. Based on our findings, which suggest a possible link between increased hippocampal volumes and a higher risk of Alzheimer’s disease, one potential explanation could be that the observed enlargement in specific hippocampal subregions reflects a compensatory mechanism in the early stages of the disease.^13,55,56^ This compensatory response might involve increased neurogenesis or synaptic reorganization that attempts to offset early pathological changes. Over time, as Alzheimer’s disease progresses, this mechanism may fail, potentially leading to the hippocampal volume reductions commonly observed in later stages of the disease. Another possibility is related to the heterogeneity in neurogenesis abnormalities observed during Alzheimer’s disease progression.^15^ Individuals with larger hippocampal volumes, which may be linked to increased neurogenesis, could be more vulnerable to the disease due to underlying genetic or physiological factors.^57^ These findings suggest that hippocampal structural changes during Alzheimer’s disease progression may be more complex and dynamic than previously understood, with potential volume increases occurring before the reductions typically observed in mild cognitive impairment or subjective cognitive decline.

In addition to elucidating the influence of hippocampal volumetric traits on Alzheimer’s disease risk, the study also identified two hippocampal volumetric traits that are reciprocally influenced by Alzheimer’s disease. A higher risk of Alzheimer’s disease was causally linked to a reduced volume of the left hippocampal body and a decreased volume in the whole left hippocampus, highlighting the progressive nature of the disease. Alzheimer’s disease is recognized for initiating neurodegenerative processes that result in the loss of neurons and synaptic connections in affected brain regions, including the hippocampus.^17,58,59^ These findings, coupled with increased volumes of hippocampal traits leading to a higher risk of Alzheimer’s disease, suggest a potential feedback loop wherein structural changes contribute to an elevated risk of Alzheimer’s disease, and the presence of Alzheimer’s disease exacerbates structural degeneration in the hippocampus. Understanding this dynamic relationship is crucial for unravelling the complexities of Alzheimer’s disease progression and may guide future research focused on developing interventions targeting both structural and pathological aspects of the condition.

The results of this study not only offer important evidence for causal relationship between hippocampal volumetric traits and Alzheimer’s disease but also offer significant insights for clinical practice and future research. The study indicates that increased volumes of the left hippocampal body, whole left hippocampus, right presubiculum head and right CA1 head are strongly linked to an elevated risk of Alzheimer’s disease, supporting the hypothesis of hippocampal volume as a potential biomarker.^13^ Therefore, regular assessment of hippocampal volume in clinical settings could help in the early detection of individuals at high risk, allowing for timely interventions to improve patient outcomes. Furthermore, our study also reveals the reciprocal influence of Alzheimer’s disease on hippocampal volume, specifically manifested as a reduction in the left hippocampal body and whole left hippocampal volume. This finding underscores the progressive nature of Alzheimer’s disease, suggesting that disease progression may lead to neuronal loss and structural degeneration of the hippocampus.^60,61^ This feedback relationship highlights the importance of understanding the interplay between structural changes and disease progression when investigating the pathological mechanisms of Alzheimer’s disease. Based on these findings, future studies should focus on how changes in hippocampal volume correlate with genetic factors and biomarkers to aid in identifying personalized intervention approaches. Additionally, longitudinal studies will help clarify the role of hippocampal volume changes at different stages of Alzheimer’s disease, providing new intervention targets for clinical practice.

### Limitations

Despite the strength of the study design and analysis, certain limitations should be acknowledged. First, since the study primarily included individuals of European ancestry, caution should be exercised when applying the findings to other populations. Second, the GWAS summary statistics for Alzheimer’s disease were used, with the 23andMe data were omitted due to access restrictions, and UK Biobank excluded to avoid sample overlap, potentially reducing statistical power. Third, despite employing various methods such as MR-PRESSO and MR-Egger to control pleiotropy or confounding bias, it is crucial to recognize these effects cannot be completely eliminated. Fourth, our analysis did not allow for an examination of potential sex differences in the causal relationship between hippocampal volume and Alzheimer’s disease risk, as sex-stratified GWAS summary statistics for Alzheimer’s disease are currently unavailable. This represents an important avenue for future research, as biological sex may have a crucial influence on the development of Alzheimer’s disease. Finally, while the MR analysis suggests causation, experimental validation is still necessary to confirm these results.

## Conclusion

In summary, our study employing an MR approach elucidates bidirectional causal links between distinct hippocampal volumetric traits and Alzheimer’s disease risk. The robustness of these findings is supported by consistent results across multiple MR methods and thorough sensitivity analysis. Our findings contribute to a deeper understanding of the intricate interplay between hippocampal structure and Alzheimer’s disease, emphasizing the pivotal role of hippocampal subfield volumes in influencing the onset and development of the disease. Future studies should aim to validate and extend these findings, employing longitudinal designs and integrating multi-modal approaches to unravel the intricate dynamics between hippocampal morphology, genetic factors and the development of Alzheimer’s disease.

## Supplementary Material

fcaf030_Supplementary_Data

## Data Availability

The data sets employed in this study are openly available. The GWAS summary statistics for the 44 hippocampal volumetric traits from UK Biobank participants can be accessed at https://open.win.ox.ac.uk/ukbiobank/big40/, while those for Alzheimer’s disease are available for download from https://ctg.cncr.nl/software/summary_statistics. This study employed following software packages, including linkage disequilibrium score regression for genetic correlation analysis (https://github.com/bulik/ldsc/) and *R* statistical software for MR analysis, utilizing the packages *TwoSampleMR* (version 0.5.6), *mr.raps* (version 0.2), *RadialMR* (version 1.0) and *MRPRESSO* (version 1.0). In addition, scripts for the present analyses are deposited at https://github.com/LiningGuo/Mendelian-randomization.
