# Supplementary material for: Causal relationships between hippocampal volumetric traits and the risk of Alzheimer’s disease: a Mendelian randomization study
Source: Brain Commun. 2025 Jan 23;7(1):fcaf030. doi: 10.1093/braincomms/fcaf030 (PMC11783321; doi:10.1093/braincomms/fcaf030)
Supplement: fcaf030_Supplementary_Data [file fcaf030_supplementary_data.docx]

**Supplementary Materials**

**Causal relationships between hippocampal volumetric traits and the risk of Alzheimer’s disease: a Mendelian randomization study**

Lining Guo^1#^, Yayuan Chen^1#^, Zuhao Sun^1#^, Jiaxuan Zhao^1^, Jia Yao^1^, Zhihui Zhang^1^, Minghuan Lei^1^, Ying Zhai^1^, Jinglei Xu^1^, Yurong Jiang^1^, Ying Wang^1^, Hui Xue^1*^, Mengge Liu^1*^, Feng Liu^1*^

^1^Department of Radiology and Tianjin Key Laboratory of Functional Imaging, Tianjin Medical University General Hospital, Tianjin, China

#Lining Guo, Yayuan Chen, and Zuhao Sun contributed equally to this work.

***Correspondence to:**

Feng Liu, Department of Radiology, Tianjin Medical University General Hospital, No. 154, Anshan Road, Heping District, Tianjin 300052, China.

E-mail: [fengliu@tmu.edu.cn](mailto:fengliu@tmu.edu.cn)

Mengge Liu, Department of Radiology, Tianjin Medical University General Hospital, No. 154, Anshan Road, Heping District, Tianjin 300052, China.

E-mail: menggeliu@tmu.edu.cn

Hui Xue, Department of Radiology, Tianjin Medical University General Hospital, No. 154, Anshan Road, Heping District, Tianjin 300052, China.

E-mail: xuehui2014@tmu.edu.cn

**Supplementary Tables**

**Supplementary Table 1. Genetic correlations between hippocampal volumetric traits and Alzheimer’s disease.**

| \| **Hippocampal volumetric traits** \| ***r_g_*** \| ***se*** \| ***z*** \| ***p*** \| \| --- \| --- \| --- \| --- \| --- \| \| Left_volume_Hippocampal-tail \| -0.1037 \| 0.0791 \| -1.3108 \| 0.1899 \| \| Left_volume_subiculum-body \| -0.1216 \| 0.1115 \| -1.0908 \| 0.2754 \| \| Left_volume_CA1-body \| -0.1437 \| 0.0829 \| -1.733 \| 0.0831 \| \| Left_volume_subiculum-head \| -0.1533 \| 0.0884 \| -1.7352 \| 0.0827 \| \| Left_volume_hippocampal-fissure \| -0.0905 \| 0.1037 \| -0.8731 \| 0.3826 \| \| Left_volume_presubiculum-head \| -0.1773 \| 0.1031 \| -1.7197 \| 0.0855 \| \| Left_volume_CA1-head \| -0.1572 \| 0.0828 \| -1.8995 \| 0.0575 \| \| Left_volume_presubiculum-body \| -0.1111 \| 0.0981 \| -1.133 \| 0.2572 \| \| Left_volume_parasubiculum \| -0.0182 \| 0.1095 \| -0.1667 \| 0.8676 \| \| Left_volume_molecular-layer-HP-head \| -0.1041 \| 0.0967 \| -1.0762 \| 0.2819 \| \| Left_volume_molecular-layer-HP-body \| -0.2037 \| 0.1138 \| -1.7895 \| 0.0735 \| \| Left_volume_GC-ML-DG-head \| -0.1285 \| 0.0814 \| -1.5782 \| 0.1145 \| \| Left_volume_CA3-body \| -0.0908 \| 0.079 \| -1.1495 \| 0.2504 \| \| Left_volume_GC-ML-DG-body \| -0.1833 \| 0.0993 \| -1.8457 \| 0.0649 \| \| Left_volume_CA4-head \| -0.1306 \| 0.0817 \| -1.5979 \| 0.1101 \| \| Left_volume_CA4-body \| -0.1748 \| 0.0928 \| -1.8833 \| 0.0597 \| \| Left_volume_fimbria \| -0.1679 \| 0.1045 \| -1.6065 \| 0.1082 \| \| Left_volume_CA3-head \| -0.1134 \| 0.0805 \| -1.4073 \| 0.1593 \| \| Left_volume_HATA \| -0.1792 \| 0.1138 \| -1.5749 \| 0.1153 \| \| **Left_volume_Whole-hippocampal-body** \| **-0.2078** \| **0.0974** \| **-2.1334** \| **0.0329** \| \| **Left_volume_Whole-hippocampal-head** \| **-0.158** \| **0.0805** \| **-1.9624** \| **0.0497** \| \| **Left_volume_Whole-hippocampus** \| **-0.1795** \| **0.0829** \| **-2.1643** \| **0.0304** \| \| Right_volume_Hippocampal-tail \| 0.0209 \| 0.0788 \| 0.2648 \| 0.7911 \| \| Right_volume_subiculum-body \| -0.0765 \| 0.0877 \| -0.8721 \| 0.3832 \| \| Right_volume_CA1-body \| -0.0443 \| 0.0808 \| -0.5476 \| 0.5839 \| \| **Right_volume_subiculum-head** \| **-0.1994** \| **0.0958** \| **-2.082** \| **0.0373** \| \| Right_volume_hippocampal-fissure \| 0.0609 \| 0.096 \| 0.6343 \| 0.5259 \| \| **Right_volume_presubiculum-head** \| **-0.1791** \| **0.0898** \| **-1.9956** \| **0.046** \| \| **Right_volume_CA1-head** \| **-0.1888** \| **0.094** \| **-2.0087** \| **0.0446** \| \| Right_volume_presubiculum-body \| -0.1627 \| 0.0966 \| -1.6842 \| 0.0921 \| \| Right_volume_parasubiculum \| -0.0194 \| 0.0948 \| -0.205 \| 0.8376 \| \| Right_volume_molecular-layer-HP-head \| -0.1093 \| 0.102 \| -1.0717 \| 0.2839 \| \| Right_volume_molecular-layer-HP-body \| -0.0925 \| 0.1016 \| -0.9107 \| 0.3624 \| \| Right_volume_GC-ML-DG-head \| -0.1457 \| 0.088 \| -1.6564 \| 0.0976 \| \| Right_volume_CA3-body \| -0.0904 \| 0.0849 \| -1.0649 \| 0.2869 \| \| Right_volume_GC-ML-DG-body \| -0.0986 \| 0.097 \| -1.0171 \| 0.3091 \| \| Right_volume_CA4-head \| -0.1259 \| 0.0893 \| -1.4107 \| 0.1583 \| \| Right_volume_CA4-body \| -0.0812 \| 0.0931 \| -0.8722 \| 0.3831 \| \| Right_volume_fimbria \| -0.1914 \| 0.1086 \| -1.7621 \| 0.0781 \| \| Right_volume_CA3-head \| -0.1415 \| 0.0883 \| -1.6017 \| 0.1092 \| \| Right_volume_HATA \| -0.1974 \| 0.1121 \| -1.7604 \| 0.0783 \| \| Right_volume_Whole-hippocampal-body \| -0.1296 \| 0.0893 \| -1.452 \| 0.1465 \| \| **Right_volume_Whole-hippocampal-head** \| **-0.1847** \| **0.089** \| **-2.0755** \| **0.0379** \| \| Right_volume_Whole-hippocampus \| -0.1407 \| 0.0831 \| -1.6926 \| 0.0905 \|   The *r_g_, se z* and *p* (two-sided) are generated from genetic correlation analysis with LDSC. The bolded font indicates that genetic correlations reached nominal significance level (*p* < 0.05). Abbreviations: CA, cornu ammonis; GC-ML-DG, granule cell layer and molecular layer of dentate gyrus; HATA, hippocampus-amygdala transition area; LDSC, linkage disequilibrium score regression; *r_g_*, genetic correlation; se, standard error. |
| --- | --- | --- | --- | --- | --- | --- | --- | --- | --- | --- | --- | --- | --- | --- | --- | --- | --- | --- | --- | --- | --- | --- | --- | --- | --- | --- | --- | --- | --- | --- | --- | --- | --- | --- | --- | --- | --- | --- | --- | --- | --- | --- | --- | --- | --- | --- | --- | --- | --- | --- | --- | --- | --- | --- | --- | --- | --- | --- | --- | --- | --- | --- | --- | --- | --- | --- | --- | --- | --- | --- | --- | --- | --- | --- | --- | --- | --- | --- | --- | --- | --- | --- | --- | --- | --- | --- | --- | --- | --- | --- | --- | --- | --- | --- | --- | --- | --- | --- | --- | --- | --- | --- | --- | --- | --- | --- | --- | --- | --- | --- | --- | --- | --- | --- | --- | --- | --- | --- | --- | --- | --- | --- | --- | --- | --- | --- | --- | --- | --- | --- | --- | --- | --- | --- | --- | --- | --- | --- | --- | --- | --- | --- | --- | --- | --- | --- | --- | --- | --- | --- | --- | --- | --- | --- | --- | --- | --- | --- | --- | --- | --- | --- | --- | --- | --- | --- | --- | --- | --- | --- | --- | --- | --- | --- | --- | --- | --- | --- | --- | --- | --- | --- | --- | --- | --- | --- | --- | --- | --- | --- | --- | --- | --- | --- | --- | --- | --- | --- | --- | --- | --- | --- | --- | --- | --- | --- | --- | --- | --- | --- | --- | --- | --- | --- | --- | --- | --- | --- | --- | --- | --- | --- | --- | --- | --- |

**Supplementary Table 2. Causal effect of hippocampal volumetric traits on Alzheimer’s disease risk.**

| **Exposure** | **Outcome** | **Method** | ***n*** | ***p*** | ***OR* (95% CI)** |
| --- | --- | --- | --- | --- | --- |
| Left hippocampal body | AD | IVW | 13 | 4.62E-04 | 1.190 (1.080～1.311) |
| Left hippocampal body | AD | MR-RAPS | 13 | 5.67E-04 | 1.193 (1.079～1.319) |
| Left hippocampal body | AD | MR-Egger | 13 | 2.73E-02 | 1.593 (1.113～2.280) |
| Left hippocampal body | AD | Weighted-median | 13 | 5.00E-02 | 1.145 (1.000～1.310) |
| Left hippocampal body | AD | Weighted-mode | 13 | 3.19E-01 | 1.132 (1.896～1.431) |
| Left whole hippocampus | AD | IVW | 12 | 7.30E-04 | 1.164 (1.066～1.270) |
| Left whole hippocampus | AD | MR-RAPS | 12 | 8.50E-04 | 1.166 (1.065～1.275) |
| Left whole hippocampus | AD | MR-Egger | 12 | 1.40E-02 | 1.470 (1.140～1.895) |
| Left whole hippocampus | AD | Weighted-median | 12 | 1.30E-02 | 1.173 (1.034～1.330) |
| Left whole hippocampus | AD | Weighted-mode | 12 | 3.48E-02 | 1.324 (1.053～1.665) |
| Right presubiculum head | AD | IVW | 4 | 4.64E-03 | 1.430 (1.116～1.832) |
| Right presubiculum head | AD | MR-RAPS | 4 | 7.75E-03 | 1.441 (1.101～1.886) |
| Right presubiculum head | AD | MR-Egger | 4 | 1.78E-01 | 3.072 (1.045～9.033) |
| Right presubiculum head | AD | Weighted-median | 4 | 1.41E-01 | 1.286 (0.920～1.796) |
| Right presubiculum head | AD | Weighted-mode | 4 | 2.19E-01 | 1.299 (0.933～1.808) |
| Right CA1 head | AD | IVW | 11 | 1.19E-02 | 1.171 (1.035～1.325) |
| Right CA1 head | AD | MR-RAPS | 11 | 2.00E-03 | 1.176 (1.061～1.304) |
| Right CA1 head | AD | MR-Egger | 11 | 6.37E-02 | 1.393 (1.024～1.895) |
| Right CA1 head | AD | Weighted-median | 11 | 3.40E-02 | 1.176 (1.012～1.367) |
| Right CA1 head | AD | Weighted-mode | 11 | 2.88E-02 | 1.351 (1.072～1.701) |

Abbreviations: AD, Alzheimer’s disease; CA, cornu ammonis; CI, confidence interval; IVW, inverse variance weighted; MR-RAPS, Mendelian randomization-robust adjusted profile score; *n*, number of instruments; *OR*, odds ratio.

**Supplementary Table 3. Causal effect of Alzheimer’s disease on hippocampal volumetric traits.**

| **Exposure** | **Outcome** | **Method** | ***n*** | ***p*** | ***β* (95% CI)** | ***se*** |
| --- | --- | --- | --- | --- | --- | --- |
| AD | Left hippocampal body | IVW | 28 | 5.27E-03 | -0.032 (-0.054～-0.010) | 0.011 |
| AD | Left hippocampal body | MR-RAPS | 28 | 5.37E-03 | -0.032 (-0.055～-0.010) | 0.012 |
| AD | Left hippocampal body | MR-Egger | 28 | 1.06E-02 | -0.042 (-0.072～-0.012) | 0.015 |
| AD | Left hippocampal body | Weighted-median | 28 | 3.54E-02 | -0.033 (-0.065～-0.002) | 0.016 |
| AD | Left hippocampal body | Weighted-mode | 28 | 2.56E-02 | -0.035 (-0.064～-0.006) | 0.015 |
| AD | Left whole hippocampus | IVW | 29 | 1.54E-02 | -0.029 (-0.053～-0.006) | 0.012 |
| AD | Left whole hippocampus | MR-RAPS | 29 | 1.08E-02 | -0.029 (-0.052～-0.007) | 0.012 |
| AD | Left whole hippocampus | MR-Egger | 29 | 4.17E-03 | -0.048 (-0.078～-0.018) | 0.015 |
| AD | Left whole hippocampus | Weighted-median | 29 | 6.10E-02 | -0.030 (-0.061～0.001) | 0.016 |
| AD | Left whole hippocampus | Weighted-mode | 29 | 2.45E-02 | -0.036 (-0.066～-0.006) | 0.015 |

Abbreviations: AD, Alzheimer’s disease; CI, confidence interval; IVW, inverse variance weighted; MR-RAPS, Mendelian randomization-robust adjusted profile score; *n*, number of instruments; *se*, standard error.

**Supplementary Table 4. Details of removed instrumental variables associated with confounders in MR analysis.**

| **Exposure** | **Outcome** | **SNP** | **chr** | **pos** | **A1** | **A2** | ***β*.exposure** | ***β*.outcome** | ***se*.exposure** | ***se*.outcome** | **eaf.exposure** | **eaf.outcome** | ***p*.exposure** | ***p*.outcome** |
| --- | --- | --- | --- | --- | --- | --- | --- | --- | --- | --- | --- | --- | --- | --- |
| Left hippocampal body | AD | rs11245388 | 10 | 126539128 | G | T | -0.061 | -0.007 | 0.008 | 0.010 | 0.569 | 0.554 | 2.05E-14 | 0.520 |
| Left hippocampal body | AD | rs2079462 | 2 | 107512320 | A | G | -0.056 | -0.015 | 0.010 | 0.012 | 0.799 | 0.795 | 1.42E-08 | 0.226 |
| Left hippocampal body | AD | rs61785580 | 1 | 46402849 | T | C | -0.090 | -0.009 | 0.015 | 0.019 | 0.072 | 0.071 | 5.34E-09 | 0.635 |
| Left hippocampal body | AD | rs6432708 | 2 | 162891848 | T | C | -0.081 | -0.010 | 0.008 | 0.010 | 0.580 | 0.613 | 7.40E-24 | 0.305 |
| Left hippocampal body | AD | rs72761269 | 5 | 66153524 | A | G | 0.046 | -0.001 | 0.008 | 0.010 | 0.360 | 0.379 | 3.28E-08 | 0.944 |
| Left whole hippocampus | AD | rs1055256 | 10 | 126446592 | G | A | -0.073 | -0.003 | 0.008 | 0.010 | 0.571 | 0.560 | 4.47E-20 | 0.736 |
| Left whole hippocampus | AD | rs2909456 | 2 | 162836954 | T | C | -0.083 | -0.016 | 0.008 | 0.010 | 0.593 | 0.623 | 4.55E-25 | 0.125 |
| Left whole hippocampus | AD | rs61785580 | 1 | 46402849 | T | C | -0.092 | -0.009 | 0.015 | 0.019 | 0.072 | 0.071 | 1.97E-09 | 0.635 |
| Left whole hippocampus | AD | rs72761269 | 5 | 66153524 | A | G | 0.048 | -0.001 | 0.008 | 0.010 | 0.360 | 0.379 | 4.72E-09 | 0.944 |
| Right presubiculum head | AD | rs1014445 | 2 | 162869203 | G | A | -0.049 | -0.013 | 0.008 | 0.010 | 0.581 | 0.610 | 8.45E-10 | 0.213 |
| Right presubiculum head | AD | rs16830444 | 2 | 199635641 | A | G | -0.046 | -0.011 | 0.008 | 0.010 | 0.354 | 0.364 | 4.88E-08 | 0.300 |
| Right CA1 head | AD | rs12472555 | 2 | 162816728 | G | T | 0.054 | 0.021 | 0.008 | 0.010 | 0.467 | 0.433 | 1.25E-11 | 0.039 |
| Right CA1 head | AD | rs2559505 | 10 | 126562724 | G | T | -0.059 | 0.001 | 0.010 | 0.012 | 0.773 | 0.761 | 1.51E-09 | 0.940 |
| Right CA1 head | AD | rs33931638 | 1 | 46500251 | A | G | -0.084 | -0.011 | 0.015 | 0.019 | 0.075 | 0.071 | 2.23E-08 | 0.546 |
| AD | Left hippocampal body | rs12590654 | 14 | 92938855 | A | G | -0.081 | -0.003 | 0.011 | 0.008 | 0.316 | 0.342 | 4.99E-14 | 0.755 |
| AD | Left hippocampal body | rs4335021 | 6 | 32386619 | T | C | 0.084 | 0.003 | 0.011 | 0.008 | 0.404 | 0.404 | 8.53E-15 | 0.729 |
| AD | Left whole hippocampus | rs12590654 | 14 | 92938855 | A | G | -0.081 | -0.002 | 0.011 | 0.008 | 0.316 | 0.342 | 4.99E-14 | 0.772 |
| AD | Left whole hippocampus | rs4335021 | 6 | 32386619 | T | C | 0.084 | 0.007 | 0.011 | 0.008 | 0.404 | 0.404 | 8.53E-15 | 0.373 |

Abbreviations: AD, Alzheimer’s disease; CA, cornu ammonis; chr, chromosome; eaf, effect allele frequency; pos, position; MR, Mendelian randomization; se, standard error; SNP, single nucleotide polymorphism.

**Supplementary Table 5. Causal associations between hippocampal volumetric traits and Alzheimer’s disease when controlling for the potential confounding factors.**

| **Exposure** | **Outcome** | **Method** | ***n*** | ***β* (95% CI)** | ***se*** | ***p*** | ***OR* (95% CI)** |
| --- | --- | --- | --- | --- | --- | --- | --- |
| Left hippocampal body | AD | IVW | 8 |  |  | 3.18E-03 | 1.233 (1.073～1.417) |
| Left hippocampal body | AD | MR-RAPS | 8 |  |  | 1.20E-03 | 1.239 (1.088～1.410) |
| Left hippocampal body | AD | MR-Egger | 8 |  |  | 4.49E-02 | 1.752 (1.134～2.706) |
| Left hippocampal body | AD | Weighted-median | 8 |  |  | 1.44E-02 | 1.261 (1.047～1.518) |
| Left hippocampal body | AD | Weighted-mode | 8 |  |  | 1.23E-01 | 1.374 (0.964～1.959) |
| Left whole hippocampus | AD | IVW | 8 |  |  | 2.06E-03 | 1.192 (1.066～1.334) |
| Left whole hippocampus | AD | MR-RAPS | 8 |  |  | 1.52E-03 | 1.196 (1.071～1.335) |
| Left whole hippocampus | AD | MR-Egger | 8 |  |  | 3.36E-02 | 1.470 (1.116～1.935) |
| Left whole hippocampus | AD | Weighted-median | 8 |  |  | 2.69E-04 | 1.320 (1.137～1.533) |
| Left whole hippocampus | AD | Weighted-mode | 8 |  |  | 3.82E-02 | 1.346 (1.071～1.691) |
| Right presubiculum head | AD | IVW | 2 |  |  | 6.64E-03 | 1.878 (1.191～2.961) |
| Right presubiculum head | AD | MR-RAPS | 2 |  |  | 1.51E-02 | 1.889 (1.131～3.157) |
| Right CA1 head | AD | IVW | 8 |  |  | 4.96E-02 | 1.164 (1.000～1.355) |
| Right CA1 head | AD | MR-RAPS | 8 |  |  | 7.41E-03 | 1.169 (1.043～1.311) |
| Right CA1 head | AD | MR-Egger | 8 |  |  | 7.43E-02 | 1.444 (1.034～2.015) |
| Right CA1 head | AD | Weighted-median | 8 |  |  | 5.72E-02 | 1.167 (0.995～1.369) |
| Right CA1 head | AD | Weighted-mode | 8 |  |  | 5.12E-02 | 1.317 (1.047～1.656) |
| AD | Left hippocampal body | IVW | 26 | -0.034 (-0.057～-0.011) | 0.012 | 3.68E-03 |  |
| AD | Left hippocampal body | MR-RAPS | 26 | -0.034 (-0.057～-0.011) | 0.012 | 3.75E-03 |  |
| AD | Left hippocampal body | MR-Egger | 26 | -0.041 (-0.071～-0.011) | 0.015 | 1.28E-02 |  |
| AD | Left hippocampal body | Weighted-median | 26 | -0.035 (-0.066～-0.004) | 0.016 | 2.75E-02 |  |
| AD | Left hippocampal body | Weighted-mode | 26 | -0.037 (-0.066～-0.009) | 0.015 | 1.75E-02 |  |
| AD | Left whole hippocampus | IVW | 27 | -0.032 (-0.056～-0.007) | 0.012 | 1.02E-02 |  |
| AD | Left whole hippocampus | MR-RAPS | 27 | -0.032 (-0.055～-0.009) | 0.012 | 6.47E-03 |  |
| AD | Left whole hippocampus | MR-Egger | 27 | -0.047 (-0.078～-0.016) | 0.016 | 6.23E-03 |  |
| AD | Left whole hippocampus | Weighted-median | 27 | -0.031 (-0.063～0.000) | 0.016 | 5.02E-02 |  |
| AD | Left whole hippocampus | Weighted-mode | 27 | -0.036 (-0.065～-0.008) | 0.014 | 1.82E-02 |  |

Abbreviations: AD, Alzheimer’s disease; CA, cornu ammonis; CI, confidence interval; IVW, inverse variance weighted; MR-RAPS, Mendelian randomization-robust adjusted profile score; *n*, number of instruments; *OR*, odds ratio; *se*, standard error.

**Supplementary Table 6. Sensitivity analyses in MR analyses.**

| **Exposure** | **Outcome** | **Heterogeneity test** | | | | **Pleiotropy test** | | | **MR-PRESSO** |
| --- | --- | --- | --- | --- | --- | --- | --- | --- | --- |
|  |  | **Method** | ***Q*** | ***df*** | ***p*** | **Egger intercept** | ***se*** | ***p*** | ***p* (global test)** |
| Left hippocampal body | AD | IVW | 10.162 | 12 | 0.602 | -0.021 | 0.013 | 0.126 | 0.617 |
|  |  | MR Egger | 7.423 | 11 | 0.764 |  |  |  |  |
| Left whole hippocampus | AD | IVW | 9.088 | 11 | 0.614 | -0.020 | 0.010 | 0.084 | 0.571 |
|  |  | MR Egger | 5.402 | 10 | 0.863 |  |  |  |  |
| Right presubiculum head | AD | IVW | 2.129 | 3 | 0.546 | -0.041 | 0.029 | 0.289 | 0.623 |
|  |  | MR Egger | 0.090 | 2 | 0.956 |  |  |  |  |
| Right CA1 head | AD | IVW | 15.013 | 10 | 0.132 | -0.013 | 0.011 | 0.260 | 0.148 |
|  |  | MR Egger | 12.937 | 9 | 0.165 |  |  |  |  |
| AD | Left hippocampal body | IVW | 22.644 | 27 | 0.704 | 0.003 | 0.003 | 0.326 | 0.698 |
|  |  | MR Egger | 21.642 | 26 | 0.708 |  |  |  |  |
| AD | Left whole hippocampus | IVW | 31.026 | 28 | 0.316 | 0.005 | 0.003 | 0.073 | 0.338 |
|  |  | MR Egger | 27.478 | 27 | 0.438 |  |  |  |  |

Abbreviations: AD, Alzheimer’s disease; CA, cornu ammonis; *df*, degree of freedom; IVW, inverse variance weighted; MR, Mendelian randomization; MR-PRESSO, MR pleiotropy residual sum and outlier; *se*, standard error.

**
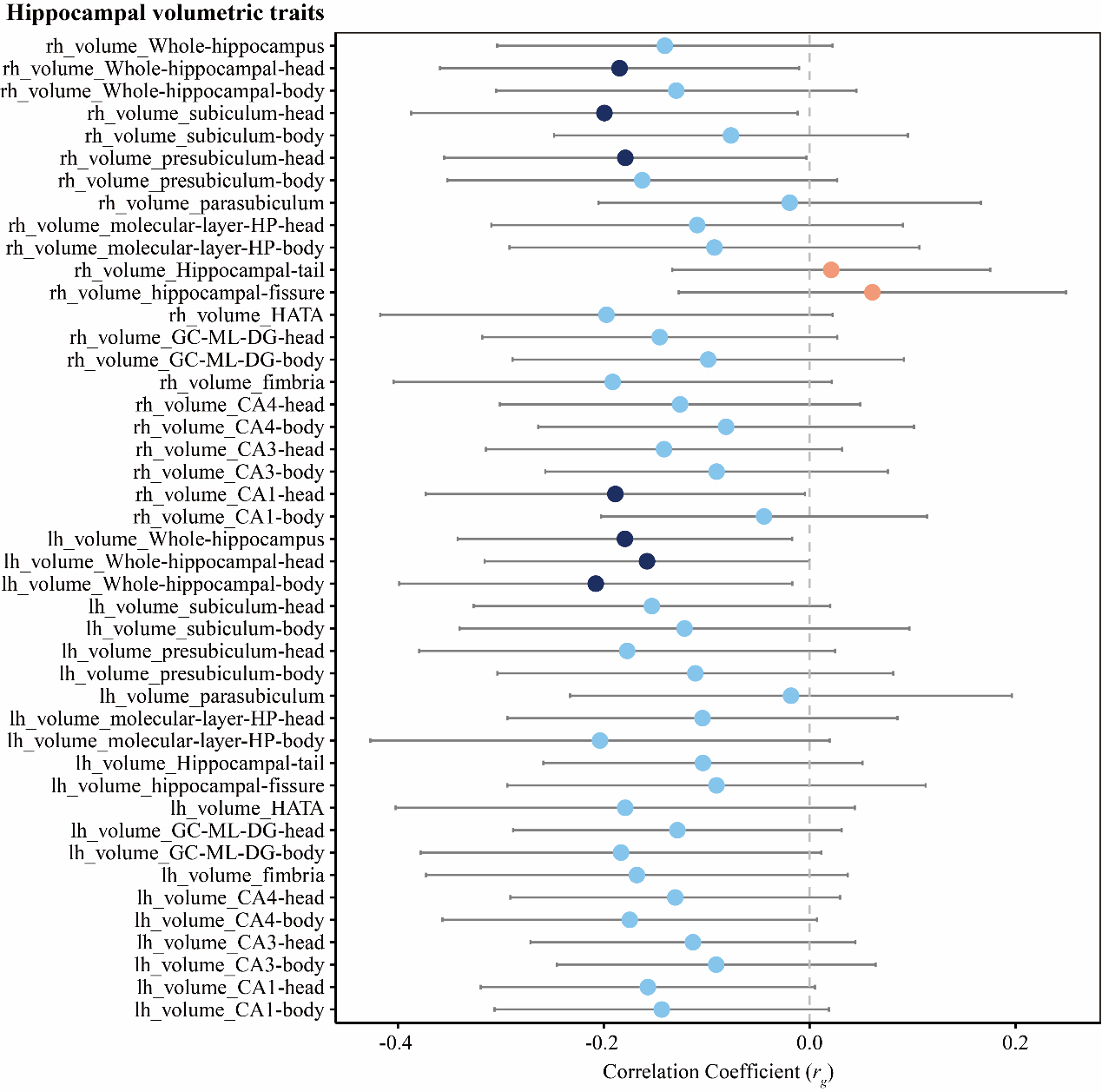
**

**Supplementary Figure 1. Genetic correlation analyses between hippocampal volumetric traits and Alzheimer’s disease.** Forest plot illustrating the genetic correlation between different hippocampal subfields and Alzheimer’s disease based on LDSC analysis. Blue points represent negative correlations, and orange points represent positive correlations. Darker blue points represent negative correlations with nominal significance (*p* < 0.05). Abbreviations: CA, cornu ammonis; GC-ML-DG, granule cell layer and molecular layer of the dentate gyrus; HATA, hippocampus-amygdala transition area; *r*_g_, genetic correlation.

**
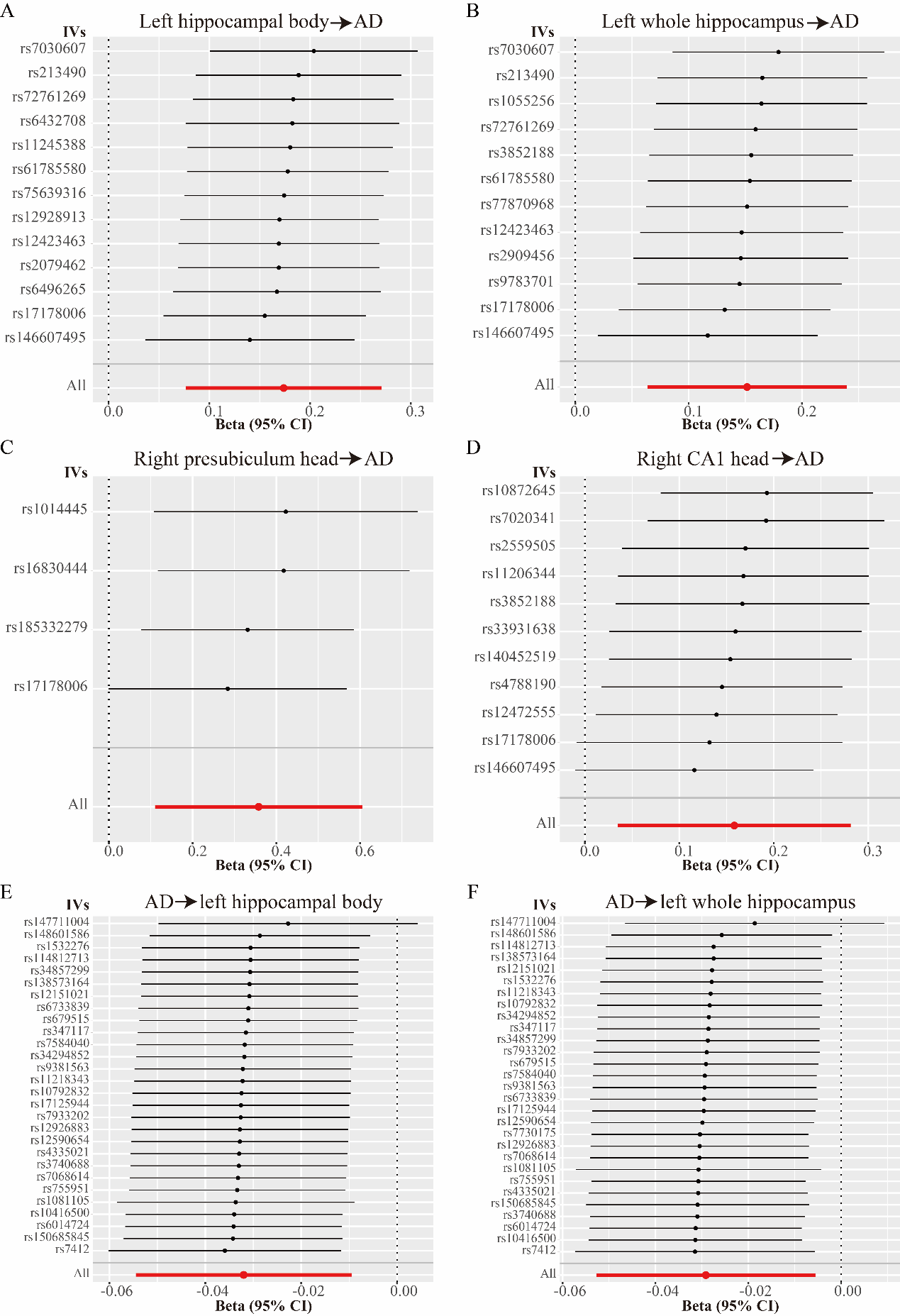
**

**Supplementary Figure 2. Leave-one-out analysis for the causal relationship between hippocampal volumetric traits (*N* = 33,224) and the risk of Alzheimer’s disease (*N*_case_ = 90,338, *N*_control_ = 1,036,225).** Leave-one-out analysis for the causal effect of volumes of (A) left hippocampal body, (B) left whole hippocampus, (C) right presubiculum head as well as (D) right CA1 head on Alzheimer’s disease risk. Leave-one-out analysis for the causal effect of Alzheimer’s disease on (E) left hippocampal body and (F) left whole hippocampus. The red points and red lines represent the BETA and 95% confidence interval in MR analyses, while the black points and black lines represent the BETA and 95% confidence interval after removing each SNP sequentially. Of note, only the IVW method was used in the leave-one-out sensitivity analysis. Abbreviations: AD, Alzheimer’s disease; CA, cornu ammonis; CI, confidence interval; IVs, instrumental variables.
